# Intermittent decoherence blockade in a chiral ring environment

**DOI:** 10.1038/s41598-021-92288-8

**Published:** 2021-06-18

**Authors:** Salvatore Lorenzo, Stefano Longhi, Albert Cabot, Roberta Zambrini, Gian Luca Giorgi

**Affiliations:** 1 Dipartimento di Fisica e Chimica, Universitá degli Studi di Palermo, Via Archirafi 36, 90123 Palermo, Italy; 2 Dipartimento di Fisica, Politecnico di Milano, Piazza L. da Vinci 32, 20133 Milan, Italy; 3 IFISC (UIB-CSIC), Instituto de Fisica Interdisciplinar y Sistemas Complejos, 07122 Palma de Mallorca, Spain

**Keywords:** Quantum information, Quantum mechanics, Qubits

## Abstract

It has long been recognized that emission of radiation from atoms is not an intrinsic property of individual atoms themselves, but it is largely affected by the characteristics of the photonic environment and by the collective interaction among the atoms. A general belief is that preventing full decay and/or decoherence requires the existence of dark states, i.e., dressed light-atom states that do not decay despite the dissipative environment. Here, we show that, contrary to such a common wisdom, decoherence suppression can be intermittently achieved on a limited time scale, without the need for any dark state, when the atom is coupled to a chiral ring environment, leading to a highly non-exponential staircase decay. This effect, that we refer to as *intermittent decoherence blockade*, arises from periodic destructive interference between light emitted in the present and light emitted in the past, i.e., from delayed coherent quantum feedback.

## Introduction

Spontaneous emission is a fundamental process in quantum optics and quantum electrodynamics^1–3^. While in the most typical cases it is described by an exponential decay of a quantum (atomic or solid state) system towards its ground state, accompanied by an irreversible emission of a photon^4^, the properties of the surrounding photonic environment^5,7^, as well as measurement^8^, or collective effects^9,10^, can largely affect spontaneous emission, with consequences ranging form control of single-photon sources to decoherence.

Dimension and geometric constraints of the photonic environment (like cavities^5^), continuous or discrete-mode structures of the reservoir^11^, as well as engineered surrounding media (for instance exhibiting band-gaps^7^), can significantly enhance or inhibit the decay rate of a single emitter. Recently, more complex photonic environments have been shown to be powerful resources for controlling light-emitter interaction in unprecedented ways^12–15^.

Coupling one or more atoms to one-dimensional chiral waveguides or topological photonic structures, that break time reversal symmetry, enables to control the directionality of spontaneous emission and to deeply modify photon-mediated interactions, with major applications in the design of integrated non-reciprocal single-photon devices, spin-photon interfaces, and in the synthesis of novel quantum states such as entangled spin states and photonic clusters states^16–23^. Likewise, ’giant’ artificial atoms, in which the atomic dimension greatly exceeds the ’photon’ wavelength and the time spent by light to cross the atom can not be neglected, provide a new paradigm of atom-field interaction^24–33^. Since the atom cannot be considered point-like anymore, spontaneous emission ceases to be exponential and the decay dynamics is described by a differential-delayed equation^25,27,30,33^, displaying strictly non-Markovian (memory) effects arising from delayed coherent quantum feedback^34–36^. Similar memory-like effects are also found in ordinary (point-like) atoms in the presence of mirrors or retardation effects^37–46^.

One among the most striking phenomena achieved through complex environment engineering is the possibility to inhibit spontaneous emission and dechoerence under certain geometric conditions, i.e. the stabilization of quantum superposition states in the presence of dissipation or other forms of decay channels or dephasing. This goal is of major relevance in different contexts ranging from quantum computation, where limiting effects of decoherence^47^ and decoherence-free-space have been broadly studied^48,49^, to quantum biology^50–52^ and quantum chemistry^53,54^, where pure dephasing and non-radiative channels are the main sources that destroy electronic coherence in molecular dynamics. Such a decoherence/decay blockade stems from the appearance of dressed light-matter states, commonly known as dark states, or else bound states in the continuum, that do not decay despite the dissipative environment. The existence of dark states and their ability to prevent quantum decay via destructive interference among different decay channels has been known since long time and studied in several areas of physics^55–74^, along with the related concept of decoherence-free subspaces^75^, i.e. regions in Hilbert space which are not affected by decoherence. A fully open question is whether spontaneous emission and decoherence can be inhibited, at least transiently or intermittently, in the absence of any decoherence-free subspace, or even though the atom-light system does not show any dark state.

In this work we show rather surprisingly that, harnessing the idea of delayed coherent quantum feedback in a reservoir with effective discrete and continuous mode structure, a point-like atom emitting in a chiral ring photonic waveguide, sustaining slow and fast counter-propagating photonic modes, undergoes intermittent decoherence suppression on a fast time scale, displaying an exotic staircase decay dynamics. Such an effect, that we refer to as *intermittent decoherence blockade*, arises from periodic destructive interference between light emitted in the present, both in fast and slow photonic modes, and light emitted in the past in the fast photonic modes. Due to the different group velocities of counter-propagating chiral modes in the ring, two different time scales are involved in the decay dynamics, which are determined by the energy level spacing in the slow and fast bands of the ring waveguide. On the fast time scale, which is of major interest in our work, the atom turns out to be effectively coupled with both a continuous set of modes (the modes of the slow ring band) into which irreversible decay and decoherence occur, and a discrete set of modes (the modes of the fast ring band), which provide delayed feedback and re-coherence in the atom and are thus ultimately responsible for the intermittent decoherence blockade predicted in our work. Clearly, on the long time scale, determined by the energy spacing between the levels in the band with the slow group velocity, the full discrete nature of the reservoir will lead to a fully coherent and unitary dynamics, and the system will almost surely return to its initial state according to the quantum recurrence theorem^76^.

## Results

### Decoherence dynamics of an atom coupled to a chiral ring

**Figure 1:**
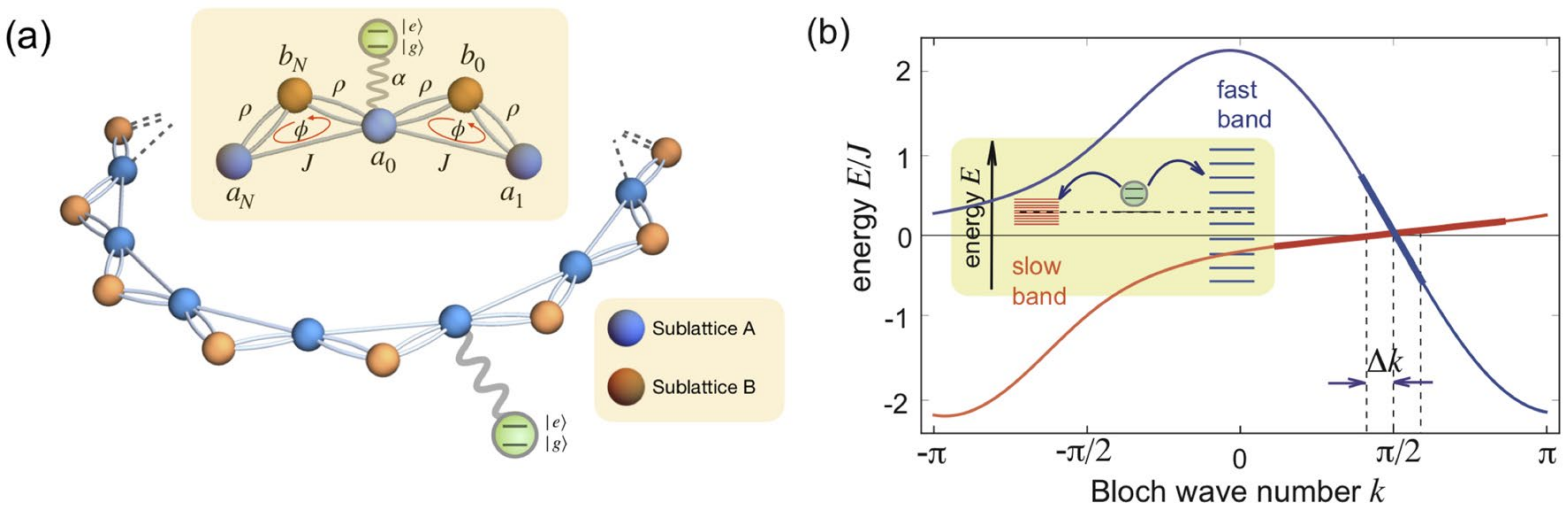
(color online) (**a**) Schematic of a two-level quantum emitter decaying on a chiral sawtooth photonic lattice. The lattice comprises two sublattices A and B, with hopping constants *J* and $\rho$ (represented by single and double lines), on a ring geometry. A synthetic magnetic flux $\phi$ is applied in each plaquette of the lattice (see the inset). (**b**) Energy diagram (dispersion curves) of the bipartite ring lattice for $\rho / J=0.5$ and $\phi =\pi /2$. Note the band closing point at $k= \pi /2$ due to the $\phi = \pi /2$ flux condition, and the existence of fast and slow bands crossing at the gap closing point, corresponding to counter-propagating modes with fast and slow group velocities $\Omega ^{\pm }$. Quantization of the Bloch wave number $\Delta k = 2 \pi /N$ due to the ring boundary conditions introduces two energy scales (i.e. energy quantization) for fast and slow bands, into which the quantum emitter decays (see the inset).

We consider the decay/decoherence dynamics of a two-level atom coupled to the radiation modes of an engineered chiral bath with broken time reversal symmetry. The photonic bath realizes a chiral sawtooth waveguide^21,22^, consisting of a bipartite lattice of cavities/resonators composed by two sublattices A and B in a ring geometry, and threaded by a synthetic gauge field $\phi$ in each plaquette, as schematically depicted in Fig. 1a. Such a model system has been investigated in some recent works and can be physically implemented in different platforms, such as squids, cold atoms, and integrated photonic circuits^21–23^. The bath is governed by the nearest-neighbor tight-binding Hamiltonian^22^

1 $$\begin{aligned} \hat{H}_B=\sum _{n=1}^N\left\{ \omega \left( \hat{a}^{\dagger }_n\hat{a}_n{+}\hat{b}^{\dagger }_n\hat{b}_n\right) +\left[ \hat{a}_{n}\left( J\hat{a}^{\dagger }_{n+1}{+} \rho e^{-i\phi }\hat{b}^{\dagger }_{n-1} +\rho \hat{b}^{\dagger }_{n}\right) +\text {h.c.}\right] \right\} , \end{aligned}$$

where $\hat{a}_n$ and $\hat{b}_n$ are the annihilation operators of the n-th *a* and *b* modes with same frequency $\omega$ (henceforth we will work in a frame rotating at $\omega$). The constants *J* and $\rho$ are the nearest-neighbour coupling between the A lattice sites and the hopping strength between the *a* and *b* modes respectively, as shown in Fig. 1a. This simple one-dimensional model admits complex couplings between lattice vertices, defining an effective magnetic flux per loop denoted by $\phi$ in Eq. (1). As detailed in Ref.^22^, the saw-tooth lattice can be realized using superconducting qubits by coupling several single-loop plaquettes, experimentally implemented in Ref.^77^. In such scenario, typical lifetimes for the tight-binding Hamiltonian modes can be extracted from the experimental measurements of Refs.^77,78^ and range from a few $\mu$s to a few tens of $\mu$s. Given the bipartite nature of the bath, the energy spectrum of $\hat{H}_B$ comprises two energy bands (Fig. 1b), with dispersion relations given by (see Methods)

2 $$\begin{aligned} E_k^{\pm }=J\cos k\pm \sqrt{J^2\cos ^2k+2\rho ^2(1+\cos (\phi +k))}. \end{aligned}$$

A non-vanishing magnetic flux $\phi$ breaks the time reversal symmetry of $\hat{H}_B$, i.e. $E_{-k}^{\pm } \ne E_{k}^{\pm }$. Remarkably, for $\phi = \pi /2$ the intraband gap closes, giving raise to a band crossing at $k=\pi /2$^22^ with energy $E_{\pi /2}^{\pm }=0$. Near the band crossing point, the dispersion relations of the two bands show an almost linear behavior, resulting in a slow ($\Omega ^+$) and fast ($\Omega ^-$) group velocities with opposite signs for the two bands, see Fig. 1b.

A point-like emitter decays into the chiral bath via a weak coupling to the radiation mode of one resonator of sublattice A. Modelling the emitter as a two level system, $\{|{g}\rangle ,|{e}\rangle \}$, with energy separation $\omega _e$, we write the free Hamiltonian of the emitter and interaction Hamiltonian with the bath as

3 $$\begin{aligned} \hat{H}_e+\hat{H}_{int}=\omega _e|{e}\rangle \langle {e}|+\alpha \left( \hat{\sigma }^-\hat{a}^{\dagger }_0+\hat{\sigma }^+\hat{a}_0\right) , \end{aligned}$$

where $\sigma ^\pm$ are the usual ladder Pauli operators. As shown in the Methods, the bath Hamiltonian can be diagonalized introducing slow and fast modes $s_k$ and $f_k$,

4 $$\begin{aligned} \hat{H}_B{=}\sum _{k}E^-_k\hat{s}^\dagger _k\hat{s}_k+E^+_k\hat{f}^\dagger _k\hat{f}_k. \end{aligned}$$

In this representation the interaction part of Eq. (3) results to be

5 $$\begin{aligned} H_{int}=\sum _{k} \frac{\alpha _k^-}{\sqrt{N}}\hat{\sigma }^+\hat{s}_k +\frac{\alpha _k^+}{\sqrt{N}}\hat{\sigma }^+\hat{f}_k + h.c. \end{aligned}$$

with couplings $\alpha _k^\pm =\alpha E^\pm _k/N^\pm _k$, where $N^\pm _k=\sqrt{(E_k^{\pm })^2 -E_k^+ E_k^-}$.

Supposing that the initial state is $|{\phi (0)}\rangle =|{e}\rangle {\otimes }|{vac}\rangle$ ($|{vac}\rangle$ denotes the vacuum state of the bath), it will evolve into $|{\phi (t)}\rangle =\varepsilon (t)|{e}\rangle {\otimes }|{vac}\rangle +\sum _{k,\pm }c_k^\pm (t)|{g}\rangle \otimes |{\psi _k^\pm }\rangle$, with $\varepsilon (0)=1$. Following standard procedures (see the Methods), we arrive at the integral differential equation for the emitter excitation amplitude

6 $$\begin{aligned} \dot{\varepsilon }(t)=-i\omega _e \varepsilon (t)-\frac{1}{N}\int _{0}^{t}\!ds \;\varepsilon (t-s) \sum _{k,\pm }\vert \alpha _k^\pm \vert ^2 e^{-iE_k^\pm s}. \end{aligned}$$

For $\phi {=}\pi /2$ the two dispersion curves (2) can be linearized near $k=\pi /2$. Assuming the emitter resonant with modes near the crossing point ($\omega _e=0$, or $\omega _e=\omega$ in the lab frame) the equation for excitation amplitude (6) becomes

7 $$\begin{aligned} \dot{\varepsilon }(t)\simeq -{\frac{1}{N}}\int _{0}^{t}\!ds\;\varepsilon (t{-}s) \sum _{k,\pm }\vert \alpha _{\pi /2}^\pm \vert ^2 e^{-i\Omega ^\pm (k-\frac{\pi }{2})s}, \end{aligned}$$

where $\Omega ^\pm {=-}J{\pm }\sqrt{J^2{+}\rho ^2}$. Note that, according to Eqs. (6) and (7), the amplitude $\epsilon (t)$ and its derivative $\dot{\epsilon }(t)$ are continuous functions of time *t*, as it should be on physical grounds, However, as discussed in the next section, for a large number *N* of sites in the ring the numerically-computed evolution of $\epsilon (t)$ displays rather sharp changes at time instants corresponding to periodic feedback from the ring. Such a scenario, which is similar to the dynamical behavior observed for a point-like two-level atom radiating in front of a mirror^37, 39, 40^, can be captured by approximating the exact integro-differential equations (6,7) with a differential-delayed equation^32, 40^. To this aim, let us note that in the large *N* limit the sum over *k* on the right hand side of Eq. (7) can be approximated by Dirac comb

$$\begin{aligned} \sum _{k}e^{-i\Omega ^\pm k s}{\sim } e^{-i\frac{N{-}1}{N}\pi \Omega ^\pm s} \frac{N}{\vert \Omega ^\pm \vert }\sum _{n=0}^{\infty }(-1)^{n(N+1)}\delta \left( s-\frac{nN}{\vert \Omega ^\pm \vert }\right) . \end{aligned}$$

In this way the time integration in (7) can be performed leading to

8 $$\begin{aligned} \dot{\varepsilon }(t){=}-\tfrac{1}{2}\gamma _0\varepsilon (t)-\sum _{n=1}^{\infty }\sum _{\pm }\gamma _n^\pm \varepsilon \!\left( t{-}nT^\pm \right) \Theta \!\left( t{-}nT^\pm \right) , \end{aligned}$$

where

9 $$\begin{aligned} \gamma _n^\pm {=}{ \frac{\vert \alpha _{\pi /2}^\pm \vert ^2 }{\vert \Omega ^\pm \vert } e^{\pm i\pi n\tfrac{N}{2}}},\quad T^\pm =\frac{N}{\vert \Omega ^\pm \vert }, \end{aligned}$$

$\gamma _0{=}\gamma _0^+{+}\gamma _0^-$, and $\Theta (x)$ stands for the Heaviside step function. From inspection of Eq. (9) we anticipate that, unless $N=2(2M)$, the damping rates exhibit a peculiar alternate change of sign at any round, increasing *n*. Note that by solving Eq. (8), the behavior of coherences is also known. In fact, the reduced density matrix of the two-level atom at time *t* is given by

10 $$\begin{aligned} {\rho }(t)=\begin{pmatrix} |\varepsilon (t)|^2\rho _{ee}&{}\varepsilon (t)\rho _{eg}\\ \varepsilon (t)^*\rho _{ge}&{}(1{-}|\varepsilon (t)|^2)\rho _{ee}{+}\rho _{gg} \end{pmatrix}\,, \end{aligned}$$

where $\rho _{jk} =\langle j|\rho (0)|k\rangle$ with $j,k=g,e$ are the entries of the possible mixed initial atomic density matrix $\rho (0)$. The first term in the right hand side of (8) represent the initial decay into “both” channels, while the successive terms take into account back excitation from the bath into the emitter when the slow and fast modes make entire loops through the ring, i.e. in the presence of delayed coherent quantum feedback. The decay dynamics is thus governed by three different time scales: (i) the decay time $T_d=1/\gamma _0$, i.e. the inverse of the decay rate $\gamma _0=\gamma _0^++\gamma _0^-$ as determined by the usual Fermi golden rule in the weak-coupling and $N \rightarrow \infty$ limits or, equivalently, by the spectral density, that is, by the Fourier transform of the sum in Eq. (6), in a master equation approach; this term is proportional to $\alpha ^{-2}$; (ii) and (iii) the feedback delay times $T^{\pm }=N /| \Omega ^{\pm }|$ for the fast and slow decay channels, depending only on bath parameters. Note that, in the limit $\rho / J \rightarrow 0$, the slow decay channel corresponds to a vanishing group velocity $\Omega ^+ \rightarrow 0$ (flat band limit), i.e. in an extremely long delay feedback time $T^+$. Here we restrict our analysis considering the decay dynamics on a time scale shorter than $T^+$, so that the slow decay channel (slow band) can be regarded as a true bath with continuous energy spectrum, into which the point emitter continuously decays. For times longer than $\sim T^-$, the discreteness of energy levels in the fast band cannot be neglected. So, on the one hand, the atom decays into a very continuum of modes (slow band), while, on the other hand, it is periodically fed by the discrete modes of the fast band, which are responsible for delayed coherent quantum feedback. For a detailed discussion about delayed quantum feedback induced by reservoirs with continuous and discrete modes see Ref.^11^.

**Figure 2:**
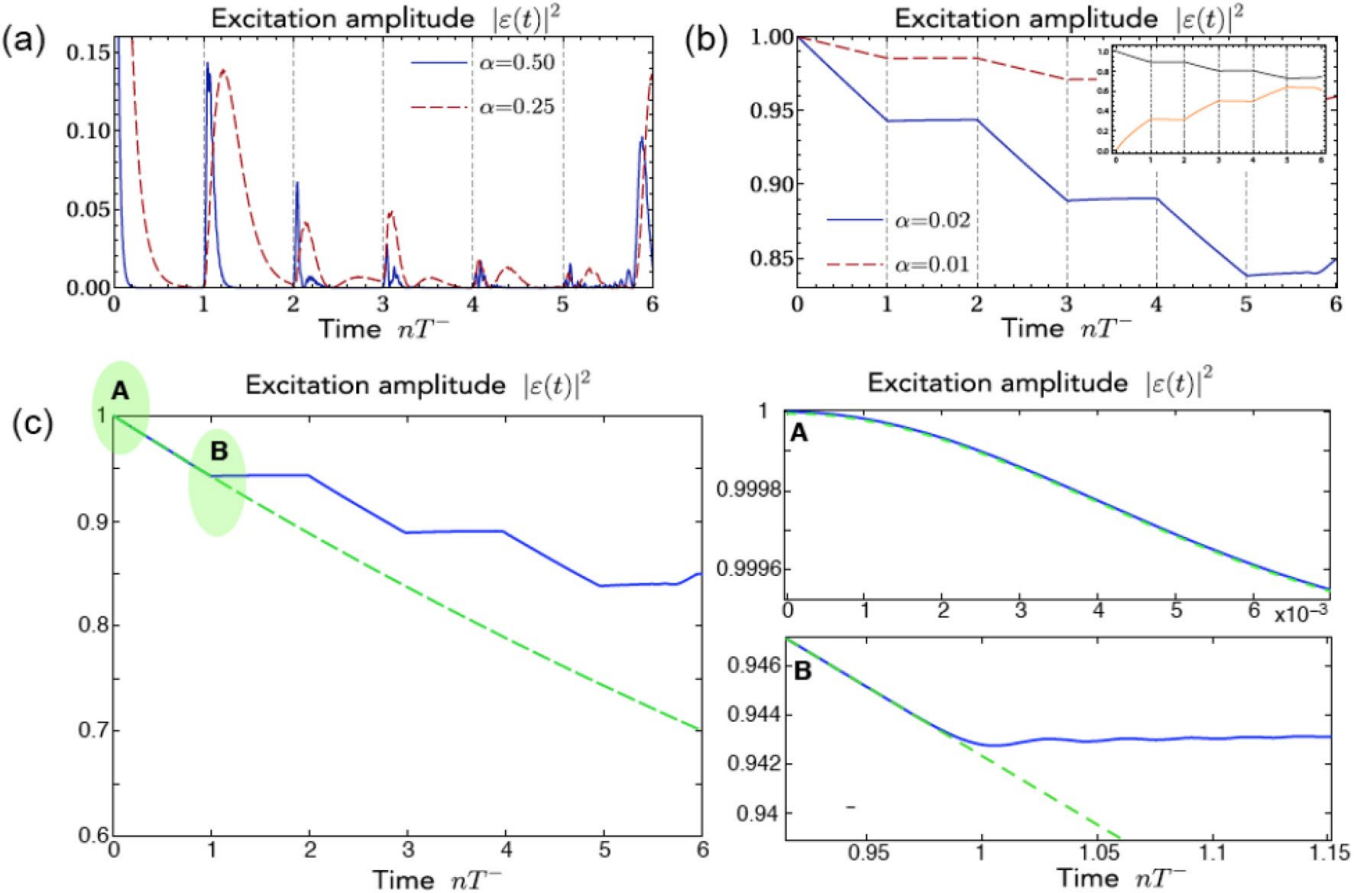
(color online) (**a**,**b**) Evolution of the excitation amplitude $|\varepsilon (t)|^2$, solution of Eq. (6) for $N=502$, $J=\rho =1$ and different values of $\alpha$. (**a**) ($\alpha =0.25$ and $\alpha =0.50$) In this regime a non exponential decay dynamics is observed, in which multiple revivals occur for $t=nT^-$. (**b**) ($\alpha =0.01$ and $\alpha =0.02$) In this regime intermittent blockade of the decay is observed until the time $t \sim 6 T^-$; at longer times feedback from the slow modes arises, which breaks intermittent decay suppression. Inset: evolution of purity (black) and von Neumann entropy (orange) for $\alpha =0.02$. (**c**) Comparison between decay dynamics for $\alpha =0.02$, $N=502$ [solid curve, as in panel (**b**)] and for $\alpha =0.02$, $N=\infty$ (dashed curve, continuous limit without delayed feedback). Note that for $0<t<T^-$ the two curves are overlapped. The plots on the right side in A and B depict an enlargement of the decay dynamics near the early time $t=0$, where the decay is parabolic (Zeno dynamics, panel A), and near the time $t=T^-$, where stopping of decoherence decay is observed in the finite *N* case due to coherent delayed feedback.

**Figure 3:**
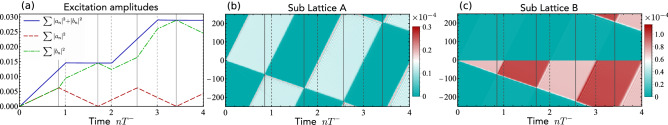
(color online) (**a**) Temporal evolution of the total excitation amplitude relative to sublattices A and B for in the intermittent decay blockade regime ($N=502$, $J{=}\rho {=}1$ and $\alpha =0.01$). (**b**),(**c**) Corresponding detailed evolution of the excitation amplitudes in sublattices A and B on a pseudocolor map (see the “Methods” for the definition of such amplitudes) . In each plot, vertical lines corresponds to times (solid) at which slow and fast waves meet and to times (dotted) at which the fast one make a complete loop.

### Intermittent decoherence blockade in a chiral bath: delayed coherent quantum feedback and staircase dynamics

According to our theoretical analysis, two very distinct dynamical scenario are expected depending on whether $T_d \ll T^-$ or $T_d \gg T^-$. While in the former case the decay dynamics shows typical multiple revivals, as observed e.g. in giant atom decay dynamics^27^, in the latter case a fully distinct behavior is observed, characterized by intermittent decays separated by intervals of decay suppression, corresponding to intermittent decoherence blockade. We computed the exact decay dynamics of $\epsilon (t)$ by numerically solving the coupled equations (15) given in the Methods, which are equivalent to the integro-differential equation (6).

An example of multiple revivals in the decoherence dynamics is shown in Fig. 2a, where the numerically-computed solution of (6) is reported for $T_d \ll T^-$ and $T^+=2\sqrt{2} +3$. Note that, after each time interval of duration $T^-$, excitation almost completely decays into the bath, however incomplete recoherence is periodically observed due quantum feedback from the fast channel into the atom. On other hand when $T_d \gg T^-$, i.e. $\alpha \ll 1$, a surprising result is observed provided that $N=2(2s+1)$ with *s* integer, namely decoherence can be intermittently suppressed (see the Methods for the dynamics with different values of *N*). An example of the decoherence dynamics in this regime obtained by simulation of the exact dynamics, Eq. (6) is shown in Figs. 2b,c and 3a. Clearly, the decay of coherence $|\epsilon (t)|$ largely deviates from an exponential decay during time $T_d$ and, most importantly, shows a nearly staircase behavior, where decoherence is inhibited at alternating time intervals of duration $T^-$, while it displays an almost linear decay outside these intervals. In the inset of Fig. 2b, we show that this peculiar behavior can be found looking at basis-independent indicators, such as the purity (defined as the trace of $\rho ^2(t)$) and the von Neumann entropy $S(\rho (t))=-{\mathrm{Tr}}[\rho (t) \log \rho (t)]$. Figure 2c compares the intermittent decoherence blockade dynamics, arising from delayed quantum feedback of the ring bath geometry, with the conventional nearly-exponential decay dynamics that one would observe in the continuous limit $N=\infty$, where the feedback is absent. For times $t<T^-$, the decay dynamics in the two cases is clearly the same. In particular, in the very early stage near $t=0$, the decay is parabolic (Zeno interval, panel A), as it should be. Likewise, in the early stage the purity dynamics shows a parabolic (Gaussian) behavior, which is not however visible on the time scale depicted in the inset of Fig. 2b. In the continuous limit $N=\infty$, after the Zeno time the decay becomes exponential with a decay rate that turns out to be in excellent agreement with the Fermi golden rule prediction $\gamma _0$. Note that, since $\gamma _0 T^- \ll 1$, the exponential decay in the range $(0,T^-)$ is very close to a linear decay. The dynamical behavior near the time $t=T^-$ is shown in panel B. As one can see, while in the continuous limit the decay is not interrupted (absence of delayed feedback from the bath), destructive interference leading to almost stopping of the decay is observed for finite *N*.

In order to better understand this intermittent decoherence blockade, one should look at the solution to (8), which can be given in terms of confluent hypergeometric functions, as shown in the Methods. In particular, the Taylor expansion of $\varepsilon (t)$ up to the second order in $\alpha$, as obtained from the solution to Eq. (8), reads explicitly

$$\begin{aligned} \varepsilon (t){\sim } {\left\{ \begin{array}{ll} 1{-}\tfrac{1}{2}\gamma _0(t{-}n T^-)\;\;\;\; \text {for} \qquad (2n)T^-{\le }\;t\;{\le } (2n{+}1)T^-\\ 1{-}\tfrac{1}{2}\gamma _0 (n+1) T^-\;\;\text {for}\;\;(2n{+}1)T^- {\le }\;t\;{\le } (2n{+}2)T^-, \end{array}\right. } \end{aligned}$$

with $n=\{0,1,2,3,\ldots \}$ such that $nT^-<T^+$. It is evident now, how the coherence decay results intermittently suppressed, for an interval of time equal to $T^-$. The intermittent decoherence blockade can be traced back to destructive interference between light emitted in the past in the fast band and light emitted in the present in both fast and slow bands of the bath, as shown in Fig. 3. The figure depicts the total excitation present in sublattices *A* and *B*. In a counter intuitive way, we see that every time the “two waves”, slow and fast, meet [this happen at times $t^*{=}n T^-T^+/(T^-+T^+)$, vertical solid lines in Fig. 3], the excitation accumulated on sublattice *A* is transferred to sublattice *B*, while the overall decay of the emitter is blocked at different alternating intervals (vertical dotted lines in Fig. 3). We emphasize that such an intermittent suppression of decoherence arising from delayed coherent quantum feedback does not require the existence of dark states, contrary to other decoherence suppression methods based on delayed feedback^34, 44, 45^. Indeed, as shown in the Methods, the full Hamiltonian $\hat{H}=\hat{H}_{B}+\hat{H}_{e} +\hat{H}_{int}$ does not sustain eigenmodes localized near the emitter.

Finally, it should be mentioned that a necessary condition for the observation of the intermittent (staircase) decoherence behavior is the resonance between the frequency of the emitter and the band crossing energy of the bath, which allowed us to perform the linearization of Eq. (7). This resonance would be lost if for instance we changed the interaction Hamiltonian, defined by Eq. (3), by considering a pure dephasing coupling of the form $\hat{H}_{\mathrm{dep}}=\alpha \hat{\sigma }_z(\hat{a}_0+\hat{a}_0^\dag )$. In this case, the decoherence caused by the bath (only observable if the non-diagonal elements of the emitter density matrix were populated at time $t=0$) would be proportional to the scalar product $\langle {vac}|e^{-i \hat{(}\hat{H}_{B}+\hat{H}_{\mathrm{dep}}^\prime ) t}|{vac}\rangle$ (where $\hat{H}_{\mathrm{dep}}^\prime =\alpha (\hat{a}_0+\hat{a}_0^\dag )$ is obtained from replacing $\hat{\sigma }_z$ with its eigenvalue 1 over the excited state of the emitter in $\hat{H}_{\mathrm{dep}}$) which would depend on the whole spectrum and would not display any sign of resonance.

## Discussion

Quantum emitters coupled to photonic waveguides represent a powerful integrated platform for complex quantum networks. While in typical scenarios the interaction between atoms and light can be treated using a master equation obeying the Born-Markov approximation, nontrivial phenomena can emerge beyond this limit. In particular, the Markovian approximation clearly fails in the presence of delayed coherent quantum feedback, where one has to take into account the effects of the finite propagation speed of light, which introduces an effective memory.

The possibility to block decoherence is generally associated with the presence of frequency gap environments or, through a less trivial mechanism, due to the presence of bound states into the continuum. In this work we showed a different scenario that exploits the chirality of the environment, which is responsible, at the same time, for the decay into a continuum of modes and delayed feedback due to a discrete set of modes. Indeed, the interference effects of delayed coherent quantum feedback enables intermittent decoherence suppression. Our results go beyond previous findings, which linked the presence of coherent quantum feedback to the emergence of dressed light-atom dark states that cause light to be trapped around the emitter, as for instance in the famous atom-in-front-of-a-mirror example. We showed that chiral waveguides can be exploited to generate nontrivial interference patterns, as they are able to separate fast and slow wavepackets, altering in this way the effect of delayed feedback. The surprising result is the total suppression of decoherence during finite time windows (whose length can be tailored modifying the system parameters) even in the absence of dark states. As an additional comment, we point out that, since the photonic environment can be readily reconfigured by tuning the gauge phase $\phi$, we can open a wide gap and fully stop the decay of the quantum emitter on demand. Moreover, since the intermittent decoherence blockade is ultimately an interference effect which is very sensitive to the gauge phase $\phi$, besides controlling decoherence our setup could be of relevance for quantum sensing. To summarize, our results suggest that chiral waveguides together with delayed coherent quantum feedback represent a powerful reservoir engineering tool of potential relevance in quantum manipulation and control, quantum sensing and quantum networks.

## Methods

### $\hat{H}_B$ diagonalization

Under periodic boundary conditions, we can introduce momentum operators, $a_k(\hat{b}_k)=1/\sqrt{N}\sum _{n=1}^Ne^{-ikn}\hat{a}_n(\hat{b}_n)$, that allow us, moving to the rotating frame at the bare energy $\omega$, to rewrite the Hamiltonian $\hat{H}_B$ in the following form

11 $$\begin{aligned} \hat{H}_B=\sum _k \begin{pmatrix} \hat{a}^{\dagger }_k&\hat{b}^{\dagger }_k\end{pmatrix} h_k\begin{pmatrix} \hat{a}_k\\ \hat{b}_k \end{pmatrix}, \end{aligned}$$

where

12 $$\begin{aligned} h_k=\begin{pmatrix} J(e^{-ik}+e^{ik})&{}g_k^*\\ g_k&{} 0 \end{pmatrix}, \end{aligned}$$

with $g_k=\rho (1+e^{-i(\phi +k)})$. Each $h_k$ can be diagonalized with its respective unitary matrix

13 $$\begin{aligned} U_k=\begin{pmatrix} E^-_k/N^-_k&{} E^+_k/N^+_k\\ g_k/N^-_k&{} g_k/N^+_k \end{pmatrix}, \end{aligned}$$

with $N_k^\pm =\sqrt{(E^\pm _k)^2+\vert g_k\vert ^2}$. In this way, the slow and fast modes $s_k$ and $f_k$ are defined as

14 $$\begin{aligned} \begin{pmatrix} \hat{s}_k\\ \hat{f}_k \end{pmatrix} =U\begin{pmatrix} \hat{a}_k\\ \hat{b}_k \end{pmatrix} =\begin{pmatrix} \frac{E^-_k}{N^-_k}\hat{a}_k+\frac{E^+_k}{N^+_k}\hat{b}_k \\ \frac{g_k}{N^-_k}\hat{a}_k+\frac{g_k}{N^+_k}\hat{b}_k \end{pmatrix}. \end{aligned}$$

### Derivation of equation for emitter excitation amplitude

An initial state of the form $|{\phi (0)}\rangle {=}|{e}\rangle {\otimes }|{vac}\rangle$ will evolve in time as

$$\begin{aligned} |{\phi (t)}\rangle =\varepsilon (t)|{e}\rangle {\otimes }|{vac}\rangle +\sum _{k,\pm }c_k^\pm (t) |{g}\rangle \otimes |{\psi _k^\pm }\rangle , \end{aligned}$$

where $\varepsilon (0)=1$, $c_k^{\pm }(0)=0$, and where $|{\psi _k^+}\rangle =\hat{s}_k^\dag | vac \rangle$, $|{ \psi _k^-}\rangle =\hat{f}_k^\dag | vac \rangle$. The time-dependent Schrödinger equation yields the set of coupled differential equations

15 $$\begin{aligned} i\dot{\varepsilon }(t)= & {} \omega _e\varepsilon (t)+\frac{1}{\sqrt{N}} \sum _{k\pm }\alpha _k^\pm c_k^\pm (t),\nonumber \\ i\dot{c}_k^\pm (t)= & {} E_k^\pm c_k^\pm (t)+\frac{1}{\sqrt{N}}\alpha _k^\pm \varepsilon (t). \end{aligned}$$

Formally integrating the latter and substituting in the former, one arrive at the integral differential equation for the emitter excitation amplitude $\varepsilon (t)$:

16 $$\begin{aligned} \dot{\varepsilon }(t)=-i\omega _e \varepsilon (t)-\frac{1}{N}\int _{0}^{t}ds \sum _{k\pm }\vert \alpha _k^\pm \vert ^2 \varepsilon (s)e^{-iE_k^\pm (t-s)}. \end{aligned}$$

### Absence of dark states

In the single-excitation sector, the energy spectrum *E* and corresponding eigenstates of the full Hamiltonian $\hat{H}=\hat{H}_{B}+\hat{H}_{e}+\hat{H}_{int}$ are obtained by solving the linear eigenvalue problem

17 $$\begin{aligned} E a_n= & {} J(a_{n+1}+a_{n-1})+ \rho b_n + \rho \exp (-i \phi ) b_{n-1} \nonumber \\&+ \alpha \epsilon \delta _{n,1} \nonumber \\ E b_n= & {} \rho a_{n}+ \rho \exp (i \phi ) a_{n-1}\nonumber \\ E \epsilon= & {} \omega _e \epsilon + \alpha a_1, \end{aligned}$$

($n=1,2,\ldots ,N$) where $\epsilon$, $a_n$ and $b_n$ are the excitation amplitudes in the atom and in the sublattices A and B, respectively. A dark state corresponds to a localized state near the emitter, the degree of localization being measured by the participation ratio $PR= (\sum _l |c_l|^2)^2 / \sum _l |c_l|^4$, where $\{c_l\}=\{a_n, b_n, \epsilon \}$ and $l=1,2,\ldots,2N+1$. For localized modes, $PR \sim 1$ while for extended states $PR \sim N$. Let us assume the atom in resonance with the electromagnetic modes of the cavities, i.e. $\omega _e=0$, and let us assume $N=2(2s+1)$ with *s* an arbitrary integer number. Numerical computation of the eigenvalues *E* of the linear system equation (17) shows that for large *N* the most localized mode, corresponding to the smallest value of the PR, is the one with energy $E=0$, and reads explicitly

18 $$\begin{aligned} a_n=0 \;, \;\; b_n=i^{n-1}, \;\;, \epsilon =- \frac{2 \rho }{\alpha }. \end{aligned}$$

apart from a normalization factor. Note that this mode does not have any excitation in sublattice A, while the excitation is distributed in the emitter and uniformly in sublattice B. The PR of this mode is given by

19 $$\begin{aligned} PR=\frac{\left[ \left( \frac{2 \rho }{\alpha } \right) ^2 +N \right] ^2 }{\left( \frac{2 \rho }{\alpha } \right) ^4+N}. \end{aligned}$$

Clearly, for large *N* one has $PR \sim N$, i.e. this mode is not localized near the emitter and corresponds to a resonance state (the ratio between the amplitudes of excitation in the emitter and in the bath scales as $\sim \rho / \alpha$, diverging as $\alpha \rightarrow 0$, which is typical of a resonance state). This means that there is not any atom-field dark state in our system.

### Solution of Eq. (8)

For $t<T^+$ we can neglect the sum over ± in Eq. (8) of the main text, i.e.

20 $$\begin{aligned} \dot{\varepsilon }(t)=-\tfrac{1}{2}\gamma _0\varepsilon (t)-\sum _{n=1}^{\infty }\gamma _n^- \varepsilon \!\left( t{-}nT^-\right) \Theta \!\left( t{-}nT^-\right) , \end{aligned}$$

with $\epsilon (0)=1$. The above equation can be readily solved in Laplace domain. After introduction of the Laplace transform $\tilde{\epsilon }(s)=\int _0^{\infty } dt \epsilon (t) \exp (-st)$, one obtains

21 $$\begin{aligned} \tilde{\varepsilon }(s)=\left( s+\frac{\gamma _0}{2}-\frac{\gamma _0^-}{e^{s T^-}+1} \right) ^{-1}, \end{aligned}$$

where we assumed $N=2(2l+1)$ with *l* integer. Expanding in power of $x=e^{sT^-}$

$$\begin{aligned} \tilde{\varepsilon }(s)=\frac{2}{2s{+}\gamma _0}+\sum _{n=1}^{\infty }4 \gamma _0^- (-1)^{n{+}1} e^{-n s T^-} \frac{(\gamma _0{-}2 \gamma _0^-{+}2 s)^{n{-}1}}{(\gamma _0{+}2 s)^{n{+}1}}. \end{aligned}$$

Returning into the time domain

22 $$\begin{aligned} \varepsilon (t)=e^{-\frac{\gamma _0t}{2}}{+} \sum _{n=1}^\infty (-1)^{n{+}1} \Theta (t{-}n T^-)\gamma _0^-(t{-}n T^-) {}_1F_{1}\left( n{+}1,2, -\frac{\gamma _0(t{-}n T^-)}{2}\right) , \end{aligned}$$

where ${}_1F_{1}$ is the confluent hypergeometric function.

**Figure 4:**
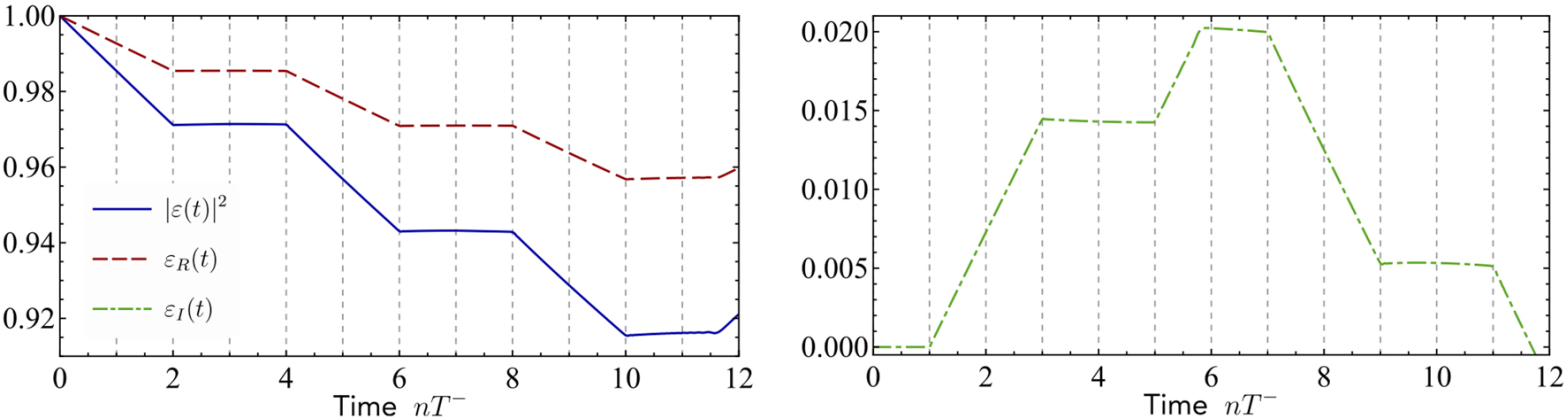
Evolution of the squared excitation amplitude $|\varepsilon (t)|^2$, solution of Eq. (6) in the main text and its real and imaginary parts $\varepsilon _R(t)$, $\varepsilon _I(t)$, for $N=501$, $J=\rho =1$ and $\alpha =0.01$. In this regime intermittent blockade of the decay is observed until the time $t \sim 12 T^-$.

### Decoherence dynamics for an odd number of sites in the ring

For the sake of completeness, let us discuss here the decay dynamics in a system with *N* odd. As discussed in the main text, the dynamics of the coherences is accurately described by equation (8). In the case of an odd number of sites per sublattices, $\gamma _n^\pm$ can take imaginary values. As a consequence, and as shown in Fig. 4, the intermittent blockade displays a doubled period which can be understood considering the approximate linearized solution for $t<T^+$. If we call $\varepsilon _R(t)$ and $\varepsilon _I(t)$ respectively the real and the imaginary part of $\varepsilon (t)$, we have

$$\begin{aligned}&\varepsilon _R(t){\sim }{\left\{ \begin{array}{ll} 1{-}\tfrac{1}{2}\gamma _0(t{-}2m T^-)\;&{} \text {for}\quad \;\;\;\;\;(4m)T^-{\le }\;t\;{\le } (4m{+}1)T^-\\ 1{-}\tfrac{1}{2}\gamma _0(t{-}2m T^-)\; &{} \text {for}\quad (4m{+}1)T^-{\le }\;t\;{\le } (4m{+}2)T^-\\ 1{-}\gamma _0(m+1) T^-\; &{}\text {for} \quad (4m{+}2)T^-{\le }\;t\;{\le } (4m{+}3)T^-\\ 1{-}\gamma _0(m+1) T^-\; &{}\text {for}\quad (4m{+}3)T^-{\le }\;t\;{\le } (4m{+}4)T^-\\ \end{array}\right. } \\&\varepsilon _I(t){\sim }{\left\{ \begin{array}{ll} \pm \gamma _0 m T^-\quad &{}\text {for}\; \;\;\;\;\;(4m) T^-{\le }\;t\;{\le } (4m{+}1)T^-\\ \pm \tfrac{1}{2}\gamma _0(t{-}(2m{+}1)T^-)\; &{} \text {for}\quad (4m{+}1)T^-{\le }\;t\;{\le } (4m{+}2)T^-\\ \pm \tfrac{1}{2}\gamma _0(t{-}(2m{+}1)T^-)\; &{} \text {for}\quad (4m{+}2)T^-{\le }\;t\;{\le } (4m{+}3)T^-\\ \pm \gamma _0 (m+1) T^-\; &{}\text {for}\quad (4m{+}3)T^-{\le }\;t\;{\le } (4m{+}4)T^-\\ \end{array}\right. } \end{aligned}$$

for $m=0,1,2,3,\dots$, where for the imaginary part of the amplitude ’$+$’ corresponds to $N=4s+1$ and ’−’ to $N=2(2s+1)+1$, with *s* integer. Thus, despite the short feedback period is still $T^-$, the dynamics displays four stages, in which the intermittency alternates between the real and imaginary parts of $\varepsilon (t)$ in periods of $2T^-$. For the parameters considered in Fig. 4, this linearized solution is accurate until $t\sim 6T^-$ for the imaginary part and $t\sim 12T^+$ for the real part, in which the first and second slow wavepackets interact with the two level system. Because of $\gamma ^+_1$ is imaginary, the slow wavepacket needs two periods to affect the real part of the amplitude, and moreover, as in this early stage of the dynamics $|\varepsilon _I(t)|\ll |\varepsilon _R(t)|$ then $|\varepsilon (t)|^2\approx \varepsilon _R(t)^2$, and the intermittent blockade of the decay in $|\varepsilon (t)|^2$ is significant until $t\sim 12T^-$ in contrast to the case studied in the main text in which it ceases at $t\sim 6 T^-$.
